# Brassinosteroid Priming Improves Peanut Drought Tolerance via Eliminating Inhibition on Genes in Photosynthesis and Hormone Signaling

**DOI:** 10.3390/genes11080919

**Published:** 2020-08-11

**Authors:** Luping Huang, Lei Zhang, Ruier Zeng, Xinyue Wang, Huajian Zhang, Leidi Wang, Shiyuan Liu, Xuewen Wang, Tingting Chen

**Affiliations:** 1 Guangdong Key Laboratory of Plant Molecular Breeding, State Key Laboratory for Conservation and Utilization of Subtropical Agro-Bioresources, College of Agriculture, South China Agricultural University, Guangzhou 510642, China; lupinghuang2019@163.com (L.H.); zhanglei@scau.edu.cn (L.Z.); ruierzeng@126.com (R.Z.); wangxinyuescau@163.com (X.W.); huajianzhang1512@gmail.com (H.Z.); wangld@mail.iap.ac.cn (L.W.); 201713070120@stu.scau.edu.cn (S.L.); 2 Department of Genetics, University of Georgia, Athens, GA 30602, USA

**Keywords:** *Arachis Hypogaea* L., brassinosteroid, drought, RNA-Seq, gene expression

## Abstract

Drought negatively affects the growth and yield of terrestrial crops. Seed priming, pre-exposing seed to a compound, could induce improved tolerance and adaptation to stress in germinated plants. To understand the effects and regulatory mechanism of seed priming with brassinosteroid (BR) on peanut plants, we treated seeds with five BR concentrations and examined dozens of physiological and biochemical features, and transcriptomic changes in leaves under well-watered and drought conditions. We found optimal 0.15 ppm BR priming could reduce inhibitions from drought and increase the yield of peanut, and priming effects are dependent on stage of plant development and duration of drought. BR priming induced fewer differentially expressed genes (DEGs) than no BR priming under well-watered condition. Drought with BR priming reduced the number of DEGs than drought only. These DEGs were enriched in varied gene ontologies and metabolism pathways. Downregulation of DEGs involved in both light perceiving and photosynthesis in leaves is consistent with low parameters of photosynthesis. Optimal BR priming partially rescued the levels of growth promoting auxin and gibberellin which were largely reduced by drought, and increased levels of defense associated abscisic acid and salicylic acid after long-term drought. BR priming induced many DEGs which function as kinase or transcription factor for signal cascade under drought. We proposed BR priming-induced regulatory responses will be memorized and recalled for fast adaptation in later drought stress. These results provide physiological and regulatory bases of effects of seed priming with BR, which can help to guide the framing improvement under drought stress.

## 1. Introduction

Drought or water deficit in soil adversely affects terrestrial crop productivity, thus threatening our food security. Most studies have focused on short-term drought responses, but few studies investigated long-term drought effects which cause agricultural loss [1,2,3]. Seasonal drought, mostly belonging to the long-term drought, is the most prevalent environmental factor affecting yield [4]. Drought triggers many phenotypic changes, physiological responses, biochemical changes, and gene expressional regulations [3,4,5]. Phytohormones, especially abscisic acid (ABA) and auxin, regulate gene expression under drought [1,2,3,6]. The expressional regulation of auxin transporter, e.g., ABCB transporters, plays important role in response to drought condition [7,8]. Engineering hormone signaling could improve plant adaptation to drought stress [9]. Epigenetic state, e.g., methylation, could affect the gene regulation in response to stress. For example, auxin homeostasis is epigenetically controlled [10].

Strategies are being employed to improve drought tolerance and adaptation in plants. Seed priming, pretreating seed before germination, is a promising approach [11]. Seed priming could improve seed germination, induce anti-stress compounds, and acclimate adaptations [11,12,13,14]. Seed priming techniques include hydro priming, hormonal priming, and redox priming etc., [14,15,16]. Among those, the hormonal priming is a widely used strategy to improve seed germination and plant growth under stresses [15], e.g., increasing germination after seed priming with auxin, cytokinin, gibberellin (GA), abscisic acid (ABA), and ethylene in pigeon pea under cadmium stress [17], and improving seed germination with ABA priming under abiotic stress conditions in *Brassica napus* [18]. 

Hormone brassinosteroids (BRs) regulate plant growth, development, and resistance to environmental stresses [19,20,21]. Exogenous BR could enhance photosynthesis, positively correlate with the tolerance to stress [20], and has been found to increase the tolerance to chilling in pepper plants [22], to heavy metal stress in *Brassica juncea* [23], to low temperature in *Brassica campestris* [24]. BR deficiency cotton mutant *pag1* resembles drought symptoms [25]. BR synthesis deficiency mutant *Micro-Tom* is susceptible to drought [26]. Different from the directly applied BR to plants, the seed priming with BR has been reported to improve tolerance under stress in later germinated plants. The seed priming with BR induced a reduced plant growth inhibitory effect of salt stress in rice [27], and increased germination rate as well as plant growth under high saline conditions in *Medicago sativa* [28]. BR binds to receptor kinase BRASSINOSTEROID INSENSITIVE1 (BRI1) to pass the signal transduction cascade, which may target hundreds of genes [21]. This makes the underlying molecular mechanisms of BR improved tolerance to stress complicated and still unclear.

Peanut (*Arachis hypogaea* L.), a member of the Fabaceae or Leguminosae family, is one of the most important oil and protein crops cultivated worldwide in tropical and subtropical regions. About 70% of the peanut plants are grown in semiarid regions, where drought is a major constraint of yield [29]. Some breeding efforts have been made to select peanut genotype with high drought tolerance [30]. The genome sequences of ancestral diploid peanuts with ~1.2 Gb genome A in *A. duranensis* or B in *A. ipaensis* [31,32], and three cultivars of *A. hypogaea* with genome AABB of 2.6 Gb are made available now [33,34,35], which facilitate genetic and genome-wide gene analysis at the molecule level. Drought stress significantly reduces pod yield and nitrogen fixation, and increases aflatoxin contamination in peanut kernels [36,37]. However, little is known about the effects of seed priming with BR on peanut plant growth and yield under well-watered and drought conditions. 

To understand the BR priming effects on peanut growth and yield under well-watered and drought conditions, we treated the peanut seeds with five different BR concentrations and then grew peanut plants under drought stress for one and two weeks, a simulated natural drought duration, with aims to check whether BR priming could improve plant tolerance to drought, which BR concentration is optimal, and what regulatory mechanisms of BR priming are involved. We examined the changes of multiple agronomic traits, yield, seed quality, photosynthesis parameters, and hormones under well-watered and drought conditions to screen for the best BR priming concentration. We found that an optimal BR priming could improve the tolerance to drought in peanut plants via reducing some inhibitory effects of drought. Genome-wide expressional regulation induced by BR priming was further investigated by using deep RNA-Seq technologies. We identified BR priming responsive genes, expressional regulation at photosynthesis and hormone signaling pathways. The study provides physiological and molecular bases of BR priming effects on peanut growth, which may guide farming improvement under drought conditions.

## 2. Materials and Methods 

### 2.1. Plant Material 

Tetraploid peanut cultivar HuaYu 39, derived from a crossbreeding between Baisha 1016 and Florunner, belongs to the pearl bean type variety with small peanut kernels. The cultivar has been widely grown for its high yield in north China. The drought resistance of this cultivar is not reported. The seeds are obtained from peanut research institute, Shandong Province, China.

### 2.2. BR Priming and Drought Treatment

Around 600 seeds were sterilized in 500 mL 1% sodium hypochlorite (NaClO) solution for 15 min followed by rinsing with distilled water for 3 min. For BR priming treatment, 300 seeds were further soaked in brassinolide, a type of Brassinosteroid, solution for 8 h at 22 °C. Five independent treatments were conducted at each BR concentration of 0 ppm, 0.05 ppm, 0.10 ppm, 0.15 ppm, and 0.20 ppm, respectively. After washing away the BR with distilled water for 3 min, every five seeds were sowed into a pot with the height of 35 cm and diameter of 40 cm full of 35 kg soil from the 0–20 cm depth of the land surface of Guangzhou (23°09′30″ N, 113°21′52″ E). Two robust seedlings were retained per pot and other seedlings were removed after 10 days. The experiment was conducted in South China Agriculture University, China (23°14′22.02″ N, 113°37′56.62″ E) during spring in 2019. 

At 32 days old, 50% of the plants had the first blossom. At this time, all pots were randomly divided into two groups for each BR concentration, 15 pots per group. One group was used as blank control with 0 ppm BR priming and kept well-watered with relative water content reaching (SRWC) ~75%. The other group were subjected to drought stress by stopping watering till the SRWC reached 40% as drought condition. The drought condition was kept SRWC 40% for seven days (7 day) and 14 days (14 day) according to previously described method [38] till sampling. Soil samples at 0–30 cm depth were harvested daily with a punch from every pot during treating period. The fresh weight of soil samples was determined then samples were oven-dried at 80 °C for 12 h. SRWC was expressed as g water g^−1^ dried soil. The soil-relative water content (SRWC) = SWC/FWC [39]. Three drought experimental replicates were conducted for each BR concentration and control, each with six biological plants. At the end of drought stress treatment, the third youngest leaf positioned from top to downward was collected from each plant in each treatment or control as one sample and stored in −80 °C freezer until subsequent analysis. At the end of 7 d and 14 d of drought treatment, all plants were well watered till peanut harvest.

### 2.3. Length and Biomass Measurement

At the end of drought stress, two plants were collected for each treatment. The length of the main stem was measured. Roots, stems, and leaves were separated and dried at 105 °C for 30 min followed by 80 °C until a constant dry weight was reached. Then dry weight of roots, stems, and leaf leaves were measured.

### 2.4. Analysis of Photosynthesis and Chlorophyll Content

A portable photosynthesis system Li-6400 (Li-COR, Lincoln, NE, USA) equipped with a red/blue LED light source was used to measure the net photosynthetic rate, intercellular CO_2_ concentration, stomatal conductance, and transpiration rate of the third leaf (usually called functional leaf in peanut plant, positioned from top to downward) between 9:00 and 11:00 A.M, and was operated using air from a large volume with a stable CO_2_ pressure. All measurements were carried out at a photo flux density of 1400 μmol m^−2^ s^−1^, the ambient CO_2_ concentration of 400 μmol mol^−1^ at 28 °C. The records were made after stable reading was achieved. The measurement was repeated for three times for each plant. The chlorophyll contents were measured on the same leaf using a chlorophyll meter SPAD-502 (Konica Minolta Sensing Inc., Osaka, Japan) [40].

### 2.5. Yield Components and Quality

After harvesting peanuts at 120 days old, the number of total pods per individual, hundred pod weight, hundred kernel weight, and shelling rate were tested as described previously [41]. Contents of oil and protein abundance in kernels were tested with a near-infrared reflectance (NIR) analyzer (DA 7250, Perten Instruments, Inc., Springfield, IL, USA) following the method reported previously [40]. 

### 2.6. Plant Hormones Analysis 

The contents of auxin, gibberellin, abscisic acid, 1-aminocyclopropane-1- carboxylate (ACC), and salicylic acid (SA) in the collected third leaves were determined as described previously [42]. 

### 2.7. RNA Extraction, mRNA Sequencing and Data Deposit

To measure the gene expression and regulation in response to drought and BR, deep short-read-based RNA-Seq sequencing was applied to measure transcriptome levels. Leaf samples from 7 d and 14 d treatments with BR priming at 0.15 ppm and two corresponding well-watered controls were selected for total RNA extraction, which was carried out with a TRIzol Reagent (Invitrogen) according to the manufacturer’s instructions. Messenger RNA (mRNA) was enriched by polyT beads from total RNAs extracted from each collected third-leaf sample and then sequenced on an Illumina platform HiSeq 4000 at Gene Denovo Biotechnology Co. (Guangzhou, China) in the paired-end 150 bp for each library followed previous procedures [43]. Three collected leaf samples from each condition were independently sequenced and 8 Gbp RNA-Seq reads were generated for each sample. The RNA-Seq reads were deposited and available at the SRA database of NCBI (https://www.ncbi.nlm.nih.gov) under the master accession number of Bioproject PRJNA629665, and the accession number for each RNA-Seq data is provided in the Supplementary File (Supplementary Table S1). 

### 2.8. Transcript Assembly, Abundance, and Pathway Analysis

All reads from RNA-Seq sequencing were cleaned and mapped into tetraploid peanut *Arachis hypogaea* cv. Tifrunner genome (version 2.0) [34] and transcripts were constructed by using HiSat2 and Stringtie as described previously [44]. The gene expression level was calculated as read count and fragment per kilobase per million reads (FPKM). Differentially expressed genes (DEGs) were examined by using DESeq2 with cutoff great than two-fold changes and *p* < 0.05 as described previously [44]. The gene ontologies (GOs) of DEGs were enriched against the background of all GOs of expressed genes in peanut (hypergeometric, *p* < 0.05). The pathways involved by DEGs were mapped into the database KEGG by using KAAS and then the pathways involved by DEGs were enriched (hypergeometric, *p* < 0.05) [44,45].

### 2.9. Statistical Analysis

One-way analysis of variance (ANOVA) was used to assess the statistically significant differences between control and treatment for the physiological and phenotypic measurements. Least significant difference test for multiple comparisons was performed for post hoc analysis at *p* < 0.05 to define statistically significant differences between means for all variables of interest. All comparative analyses were performed with IBM SPSS statistics (version 24).

## 3. Results

### 3.1. BR Priming Effects on Plants Height and Biomass of Peanut under Well-Watered and Drought Conditions

**Figure 1:**
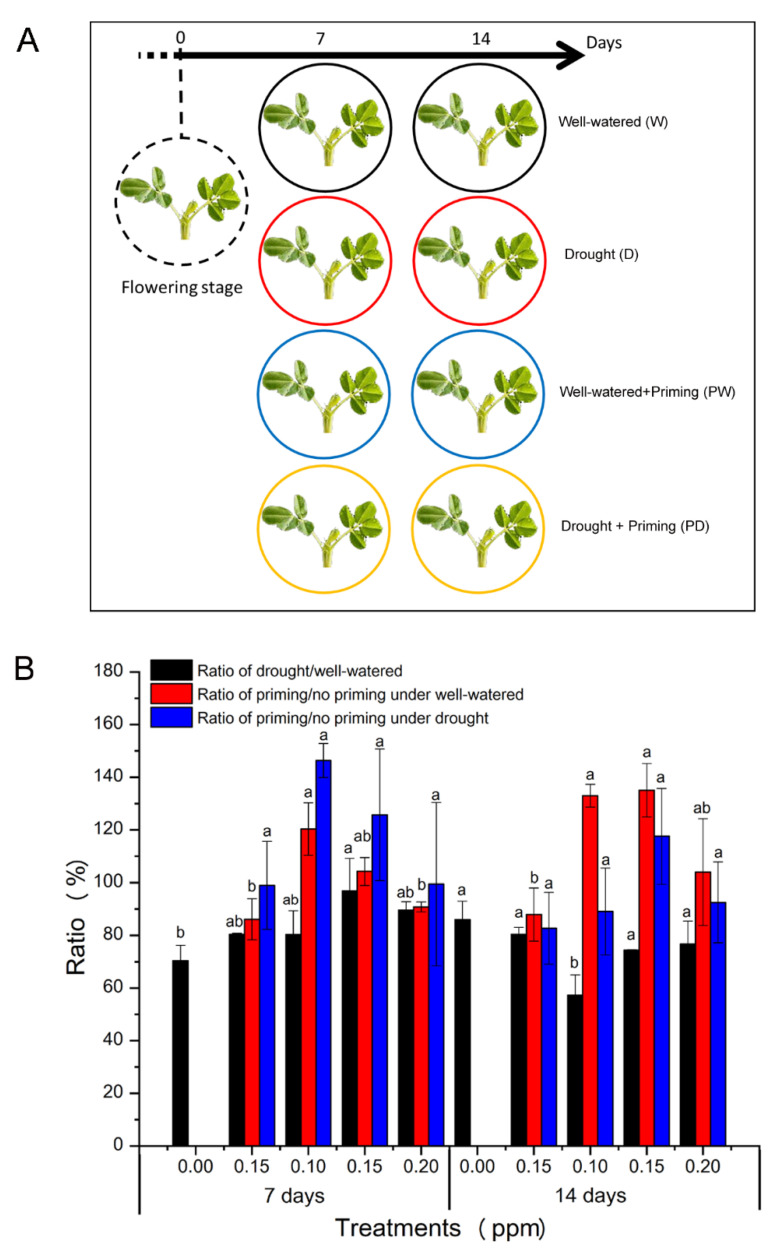
Effects of seed priming with brassinolide on plant height of peanut under well-watered and drought conditions. 32-day-old peanut plants were grown from seeds with or without brassinolide solutions priming at 0 ppm as the blank control, 0.05 ppm, 0.10 ppm, 0.15 ppm, 0.20 ppm. (**A**) A diagram scheme showing that the plants at in florescence stage were treated with drought or well-watered condition for 7 d or 14 days. (**B**) Relative ratio of plant height. Three experiments were conducted, each with six plants. Letters represent the statistically significant difference between ratios (*p* < 0.05) as determined by the least significant difference test at 7 days and 14 days, respectively. Data represent the mean ± standard error from three replicated experiments.

To investigate the effects of seed priming with BR on peanut plants, we soaked peanut seeds with BR solutions and treated the control group without BR. A peanut cultivar Huayu 39, widely grown in China, was used in this study. In total, five BR concentrations at 0 ppm as a no BR control, 0.05 ppm, 0.10 ppm, 0.15 ppm, 0.20 ppm were applied as BR priming at the seed stage. 32-day-old plants were started to be kept under a well-watered condition with soil relative water content (SRWC) 75% or drought conditions with SRWC 40% for 7 days and 14 days. After drought treatments, all plants were watered normally till harvested. To analyze the BR priming effects on plant growth, we measured plant height and calculated the relative change of plant height, defined as the ratio of plant height under drought to that under well-watered condition. Comparisons showed the ratio was less than 1, meaning that drought inhibits plant growth, and was increased a little with the increase of BR concentration (Figure 1 and Supplementary Table S2). Further analyses of the priming ratio, as defined the ratio of plant height with priming to that without priming at the same time point, revealed a higher priming ratio under drought than that under corresponding well-watered condition after 7-d treatment; however, the ratio varied after 14-d treatment (Figure 1 and Supplementary Table S2). BR priming at 0.15 ppm produced the significantly (*p* < 0.05) highest ratio, meaning the least inhibition, in both 7-d and 14-d treatments. Together, BR priming reduces drought inhibition of growth and promotes plant growth under short-term drought stress while the promotion was dependent on the duration of drought stress. The optimal BR priming concentration was between 0.10 ppm and 0.15 ppm. 

Comparisons of dry weight of roots (DWR), stems (DWS), and leaves (DWL) showed that drought significantly reduced (*p* < 0.05) the biomass of DWR, DWS, and DWL compared with those under well-watered conditions, no matter with or without BR priming. With BR priming, the reduction in biomass was minimized. Among the tested concentrations, 0.1 and 0.15 ppm BR priming had the least reduction of biomass under drought among all BR priming concentrations (Supplementary Table S3). 

### 3.2. Biochemical and Physiological Changes in Photosynthesis Process Induced by Seed BR Priming

**Figure 2:**
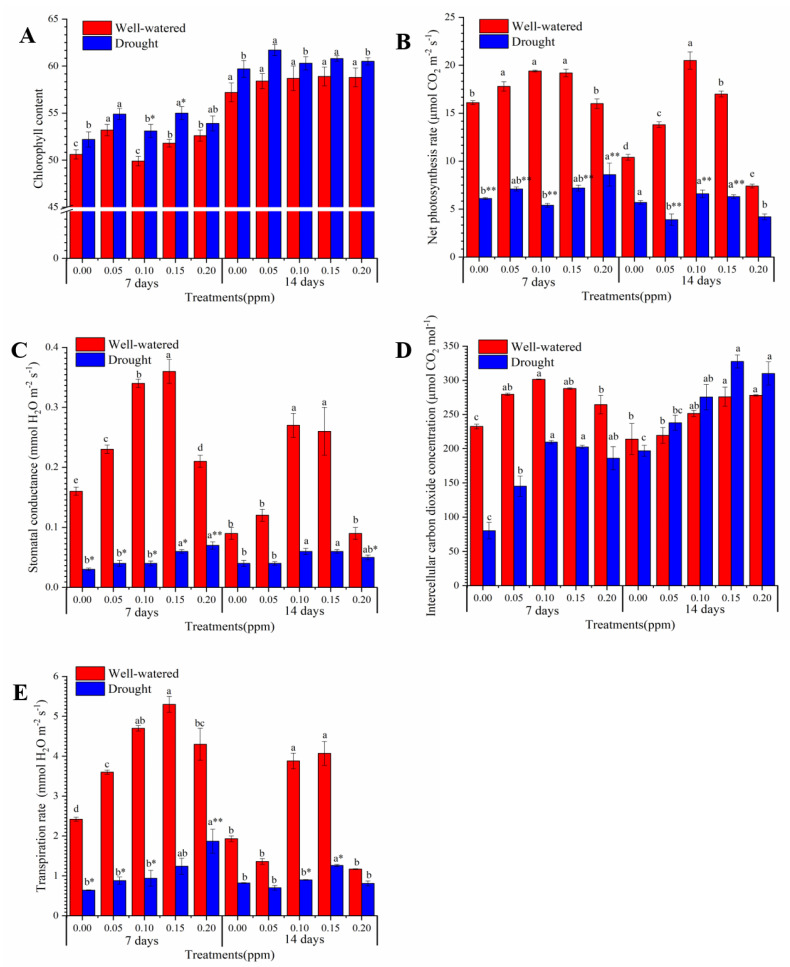
Physiological changes in photosynthesis induced by brassinolide priming under well-watered and drought conditions. (**A**) Chlorophyll content; (**B**) Net photosynthesis rate; (**C**) Stomatal conductance; (**D**) Intercellular carbon dioxide concentration; (**E**) Transpiration rate. Data are presented as mean ± standard error (*n* = 3). Different letters above the bar indicate the statistically significant difference among the five brassinosteroid (BR) priming at 7 days of drought treatments and 14 days of drought treatment, respectively as determined by the least significant difference test. * and ** represent the statistically significant difference at *p* < 0.05 and *p* < 0.01, respectively, between drought treatment and well-watered treatment. BR priming concentrations were set to 0.00, 0.05, 0.10, 0.25, and 0.20 ppm. 7 days, 7 days of treatment; 14 d, 14 days of treatment.

To investigate the effects of BR priming on photosynthesis in leaves, we measured the chlorophyll content, photosynthetic rate (Pn), intercellular carbon dioxide concentration (Ci), stomatal conductance (Gs), and transpiration rate (Figure 2). Under the well-watered condition, BR priming increased chlorophyll content at 0.05 ppm, 0.15 ppm, and 0.20 ppm except 0.10 ppm BR priming after seven days (7 d) of treatment compared with that at the 0.00 ppm control; but there was no difference in chlorophyll content between 0.00 ppm BR priming control and other BR priming samples after 14 days (14 d) of treatment (Figure 2A). Under drought condition, 0.10 ppm and 0.15 ppm BR priming increased the chlorophyll content significantly (*p* < 0.05) under 7-d drought compared with that in corresponding well-watered condition, while no significant change was observed at other BR concentrations. With BR priming, the net photosynthetic rate was decreased significantly (*p* < 0.05) in leaves in response to 7-d drought stress compared with that at well-watered condition in all tests; the net photosynthetic rate was decreased significantly in samples with 0.05 ppm, 0.10 ppm, and 0.15 ppm BR priming in response to 14-d drought treatment compared with that at well-watered condition (Figure 2B). After 7-d drought treatment, the Ci in all samples were decreased, although not significant, compared with those under well-watered condition, and a greater decreasing was found in samples with no BR priming than with BR priming. After 14-d drought treatment, the Ci was increased with BR priming under drought, though not statistically significant, compared with that under well-watered condition; however, the Ci was decreased in 0.00 ppm BR priming sample compared with that in control (Figure 2D). Among drought stress samples, Ci was increased significantly (*p* < 0.05) with BR priming, with the maximum increase at 0.10 ppm and 0.15 ppm priming, compared with that 0.00 ppm BR priming in both 7-d and 14-d treatments. Together, these results suggest that Ci under drought stress could be partially rescued or increased by BR priming. Stomatal conductance was significantly reduced (*p* < 0.05), three- to five-folds lower in most tests, under drought relative to the corresponding well-watered condition at the same time point. Within drought stresses treated samples, 0.15 ppm BR priming significantly increased (*p* < 0.05) the Gs, but Gs was still much lower than that in well-watered condition, compared with that without BR priming after 7-d or 14-d treatment, meaning 0.15 ppm BR priming only slightly buffered the drought effect on Gs (Figure 2C). Without BR priming, the transpiration rate was significantly reduced by drought compared with that in well-watered condition in the 7-d treatment only. With BR priming, Tr was still reduced by drought compared with that by well-watered condition and the significant reduction was found at 0.05 ppm, 0.10 ppm, and 0.20 ppm BR priming after 7-d drought treatment, and at 0.10 ppm and 0.15 ppm priming after 14-d drought treatment (Figure 2E). Together, BR priming with optimal concentration ~0.15 ppm could partially rescue the inhibition of drought stress on photosynthesis.

### 3.3. BR Priming Effects on Yield Components and Quality of Peanut Under Well-Watered and Drought Conditions

**Figure 3:**
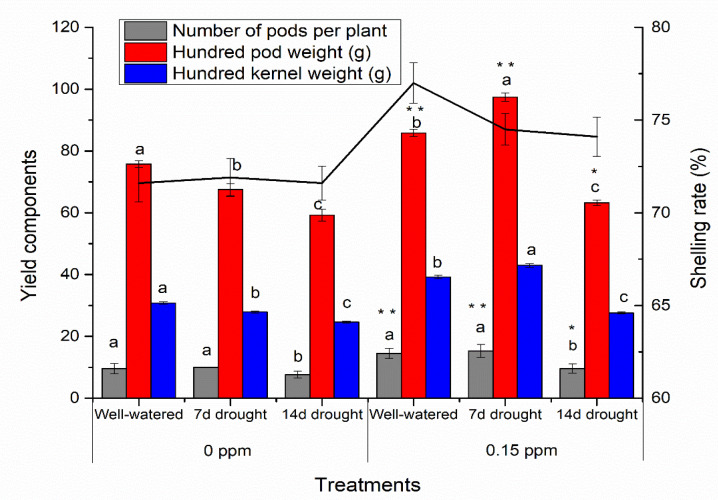
Effects of BR priming on peanut yield components under well-watered and drought conditions. Data are presented as the mean ± standard error (*n* = 3). Letters represent the statistically significant difference between any two conditions of well-watered, 7-d drought, and 14-d drought (*p* < 0.05) as determined by the least significant difference test at the same ppm of concentration. Asterisk * and ** represent the statistically significant difference at *p* < 0.05 and *p* < 0.01, respectively, between 0.15 ppm BR priming and 0 ppm BR priming. 7 d, 7 days; 14 d, 14 days.

To examine the BR priming effects on the yield components, the number of pods per plant (NPP), hundred pod weight (HPW), hundred kernel weight (HKW), and shelling rate (SR) were measured in 0 ppm and 0.15 ppm BR priming experiments under drought or well-watered conditions (Figure 3 and Supplementary Table S4). Under well-watered condition, NPP, HPW, HKW, and SR were significantly increased (*p* < 0.05) by 51%, 13%, 27%, and 8%, respectively, in plants with BR priming compared with those without BR priming, suggesting an increase with BR priming. With 7 days of drought stress, NPP, HPW, HKW, and SR were significantly increased (*p* < 0.05) by 53%, 44%, 54%, and 4%, respectively, in plants with BR priming compared with those without BR priming. With 14 days of drought stress, NPP, HPW, HKW, and SR were significantly increased (*p* < 0.05) by 26%, 7%, 12%, and 3% compared with those without BR priming. However, the increase of yield components in plants with 14-d drought treatment was much lower than that with 7-d drought treatment (Figure 3 and Supplementary Table S4). The protein content and oil content in peanut kernels were not changed between BR priming and no priming treatment, although the protein and oil content were affected with 14-d drought plus 0.15 BR priming compared with those with well-watered condition plus 0.15 BR priming (Supplementary Table S5). 

### 3.4. BR Priming Effects on Hormone Contents in Peanut Leaves under Well-Watered and Drought Conditions

**Figure 4:**
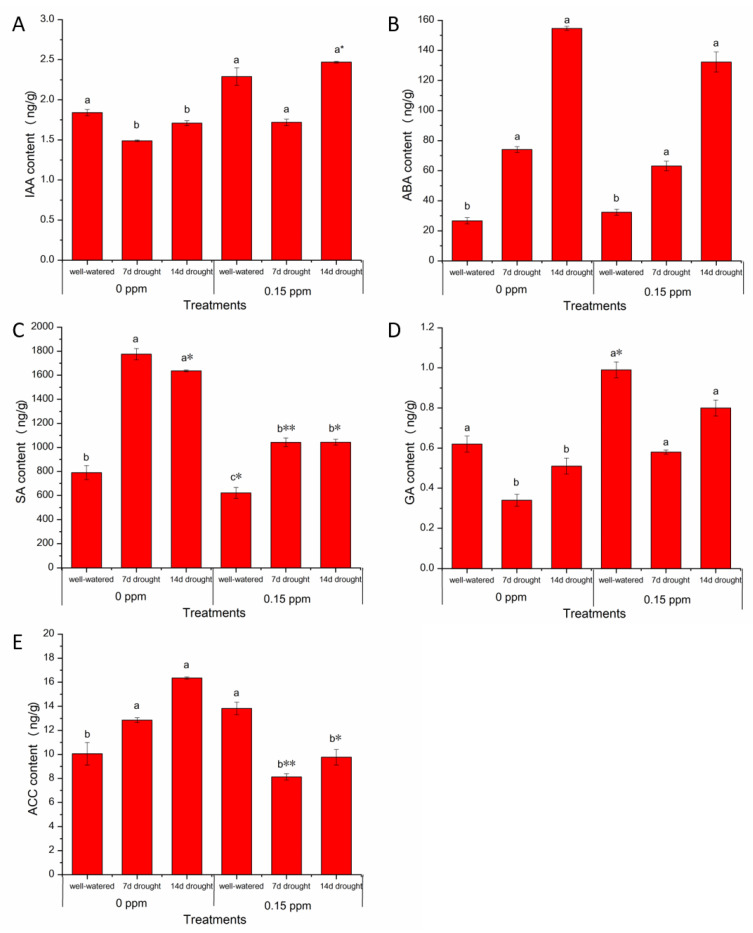
Effects of seed priming with brassinolide on hormone contents in peanut leaves under well-watered and drought conditions. (**A**) IAA content; (**B**) ABA content; (**C**) SA content; (**D**) GA content; (**E**) ACC content. Data are presented as the mean ± standard error (*n* = 3). Letters above the bar represent the statistically significant difference between any two conditions of well-watered, 7-d drought, and 14-d drought (*p* < 0.05) as determined by the least significant difference test. Asterisk * and ** represent the statistically significant difference at *p* < 0.05 and *p* < 0.01, respectively, between 0.15 ppm BR priming and 0 ppm BR priming. 7 d, 7 days; 14 d, 14 days. IAA, indole-3-acetic acid; GA, gibberellin; ABA, abscisic acid; SA, salicylic acid; ACC, 1-aminocyclopropane-1-carboxylic acid as the precursor of ethylene.

Since plant hormone signaling could crosstalk [2], we checked the change of five hormones in response to BR priming. Without BR priming, a significant increase (*p* < 0.05) was observed under drought compared with under well-watered condition for defense associated hormones abscisic acid (ABA), salicylic acid (SA), and 1-aminocyclopropane-1-carboxylic acid (ACC) which is the direct precursor for ethylene (Figure 4), while a significant decrease was found in growth-promoting hormones indole-3-acetic acid (IAA) and gibberellin (GA) under drought compared to those with well-watered condition. Similar changes in hormones were reported under drought in other crops [3].

With 0.15 ppm BR priming, the levels of IAA and GA were the same as those under well-watered condition. Compared with no BR priming, a trend of increase of IAA was found with 0.15 ppm BR priming and was significant after 14-d drought treatment (Figure 4A), which suggested BR priming buffered changes of IAA and GA induced by drought to a normal level. With BR priming, significantly increased (*p* < 0.05) levels of ABA and SA were observed under drought compared with those with the same priming under well-watered conditions. The SA levels were significantly higher in priming samples than those in no-priming samples, indicating that BR priming increased high levels of SA (Figure 4C). With 0.15 ppm BR priming, the ACC levels were reduced under drought compared with those under the well-watered condition (Figure 4E). ACC levels were significantly decreased (*p* < 0.05) with BR priming compared with that without BR priming under drought conditions. 

### 3.5. Genome-Scale expreSsion Profiles and DEGs in Response to BR Priming under Well-Watered and Drought Conditions

**Figure 5:**
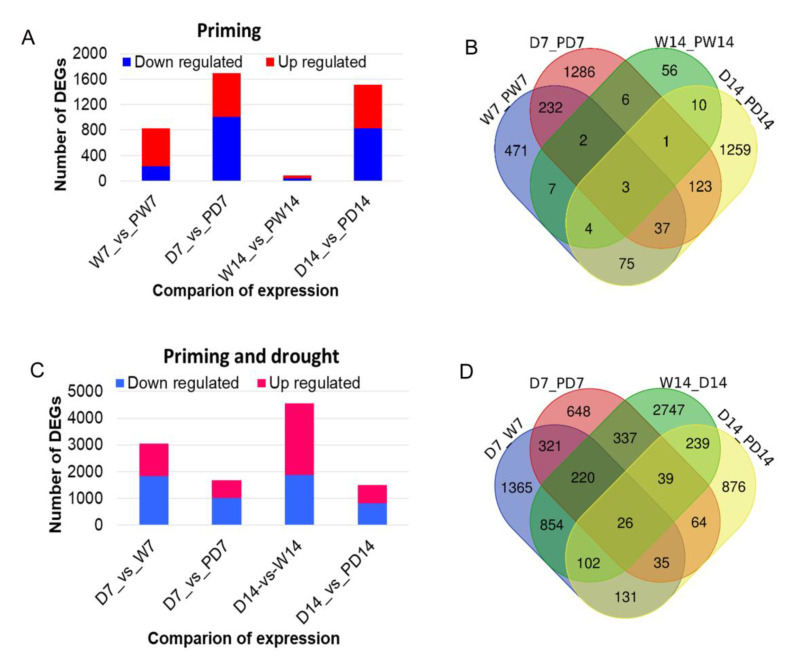
Differentially expressed genes induced by brassinolide priming in peanut leaves under well-watered and drought conditions. (**A**) The number of DEGs induced by BR priming; (**B**) Venn graph showing the common and specific DEGs induced by BR priming; (**C**) The number of DEGs induced by combined BR priming and drought; (**D**) Venn graph showing common and specific DEGs induced by combined drought and BR priming. D7_vs_w7: comparison between 7 days of drought and 7 days of well-watered condition; D7_vs_PD7: comparison between 7 days of drought and combination priming and 7 days of drought; D14_vs_w14: comparison between 14 days of drought and 14 days of well-watered condition; D14_vs_PD14: comparison between 14 days of drought and combination priming and 14 days of drought.

To understand the regulation of responsive genes, we measured the transcriptome in the third youngest leaf samples of six plants in triplicate experiments from conditions with 0.15 ppm or without BR priming under drought or well-watered control using deep RNA-Seq technology on an Illumina HiSeq 4000. ~8 Gbp reads for each sample were generated, cleaned, and mapped into reference genome assembly of peanut cultivar Tifrunner [34] with HiSat2, and then transcripts were built with Stringtie [46], followed by checking differentially expressed genes with DESeq2 [47] using previously described parameters [44]. In total, we detected 71,520 expressed genes by RNA-Seq analysis from all samples, which is close the transcriptome atlas consisting of ~80,000 transcripts from multiple peanut tissues [48]. Under well-watered conditions, 880 and 83 DEGs were identified in leaves samples from PW7 and PW14 relative to those from W7 and W14 without BR priming at the same time point, respectively. Among these, more DEGs were up-regulated than down-regulated (Figure 5). Under drought conditions, 1689 and 1511 DEGs were identified in leaves from DP7 and DP14, with BR priming at 7- and 14-day stages, relative to those from D7 and D14 without BR priming at the same time point, respectively. This revealed that BR priming induced more DEGs under drought conditions than well-watered conditions (Figure 5A). Further comparisons identified 18 commonly induced DEGs between 7-day and 14-day stages under the well-watered condition, and 274 commonly induced DEGs between 7-day and 14-day treatments with drought (Figure 5B), whose expression should be constantly affected by BR priming. To investigate the combined effects of both drought and BR priming, we identified 3054 DEGs, 1829 up-regulated, and 1225 down-regulated, induced by drought-only from comparison W7 vs. D7, which was much more than the 1689 DEGs from comparison D7 vs. PD7 with combined treatments of drought and priming, indicating BR priming reduced by 55% of the number of DEGs induced by drought stress only (Figure 5C). Similarly, the number of DEGs from comparison D14 vs. PD14 with combined effects of drought and priming was 33% of that from comparison D14 vs. W14 with drought effect only. Further comparisons revealed that 602 and 406 DEGs were shared by both drought effect and BR priming effect after 7-d and 14-d treatments (Figure 5D). Together, the results suggested that BR priming could greatly reduce the number of induced DEGs under drought treatments.

### 3.6. Gene Ontology of BR-Priming Induced DEGs under Well-Watered and Drought Conditions

**Figure 6:**
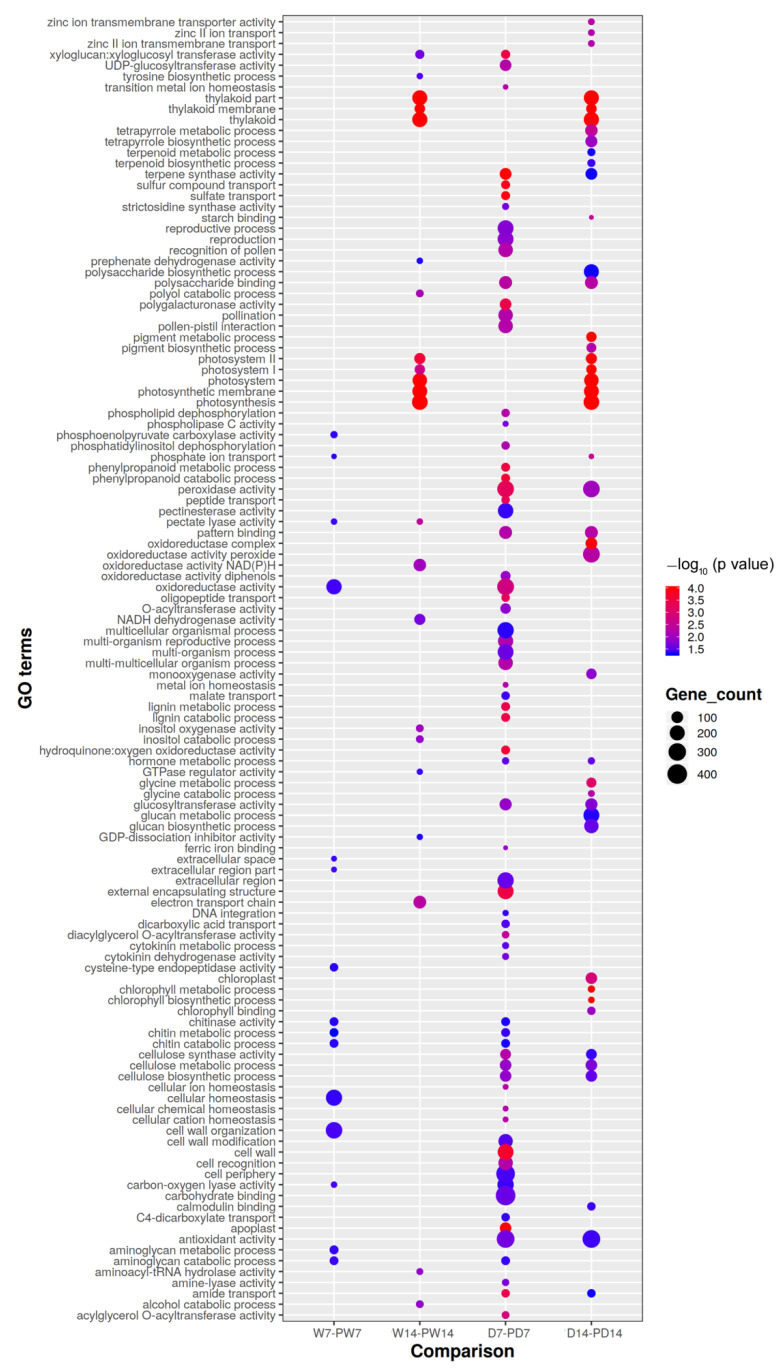
Comparison of GOs of DEGS induced by brassinolide priming under well-watered and drought conditions. Image showing the top 150 enriched gene ontology (GOs) of differentially expressed genes (DEGs) induced by seed priming with BR. GO enrichment was conducted against all GOs in peanut with statistical significance (hypergeometric test *p* < 0.05). W7, PW7, W14, PW14, D7, PD7, D14, and PD14 represent the samples, where 7, 14, W, D, and P represent after 7 days of treatment, after 14 days of treatment, under well-watered condition, under drought condition and seed priming with BR, respectively. Gene_count represents the number of DEGs.

To further understand the functions of induced DEGs by BR priming, gene ontology (GO) enrichment analysis (hypergeometric test, *q* < 0.05) was conducted based on the annotation of genes in reference genome of peanut. We found 94, 22, 62, and 35 enriched GOs of priming induced DEGs from comparisons of D7 vs. PD7, W7 vs. PW7, D14 vs. PD14, and W14 vs. PW14, respectively. Investigation of the distribution of these enriched GOs revealed that the GOs enriched under well-watered conditions were mostly not overlapped by those from drought conditions although some GOs were overlapped such as photosynthesis-related GOs (Figure 6). Few GOs of DEGs were overlapped between 7 d and 14 d well-watered conditions. However, more common GOs were identified under drought treatments than under well-watered conditions. GOs of thylakoid, polysaccharide binding, glucosyltransferase activity, antioxidant activity, and amide transport were shared by both 7-d and 14-d drought treatments (Figure 6). 

### 3.7. Enriched Pathways of DEGs Induced by BR Priming under Well-Watered and Drought Conditions

We further examined the pathways involved by DEGs which were induced by BR priming under well-watered and drought treatments. After mapping DEGs against the pathway database KEGG, 10, 12, 39, and 30 pathways were identified as significantly changed (*p* < 0.05, hypergeometric test) in BR-priming samples of PW7, PW14, PD7, and PD14 relative to those without priming at the same time point, respectively. More pathways involved by the BR priming were enriched in drought conditions than in well-watered conditions, which may be induced by integrated effects of both BR priming and drought. Comparisons revealed that ten enriched pathways were commonly regulated under drought conditions of both PD7 and PD14 (Table 1). These pathways included mRNA surveillance pathway for eliminating abnormal mRNAs, porphyrin, and chlorophyll metabolism for photosynthesis, nitrogen metabolism, and zeatin biosynthesis for hormone cytokinin (Table 1). However, no shared pathway was identified between PW7 and PW14, suggesting that enriched pathways in each well-watered condition were dynamically induced with BR priming.

### 3.8. Regulations on BR-Priming Inducing DEGs in Photosynthesis Pathway 

**Figure 7:**
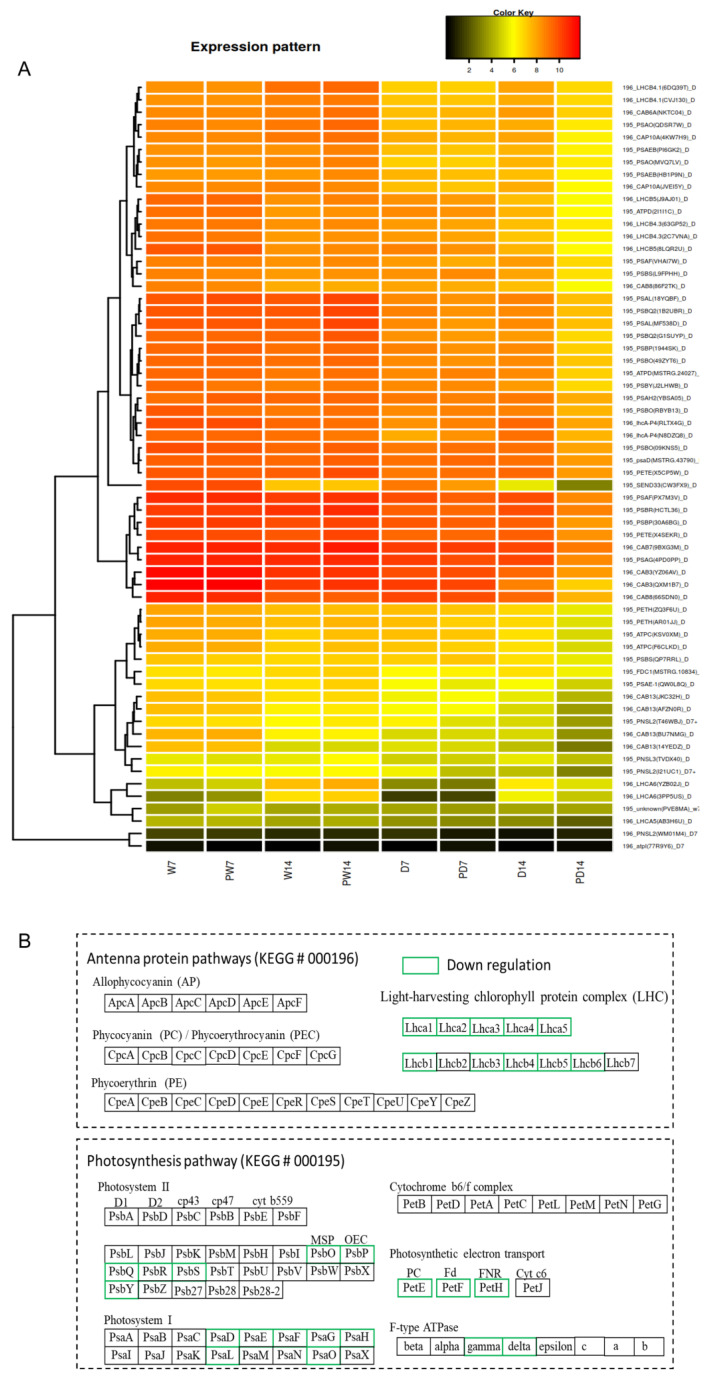
Comparison of DEGs in photosynthesis-associated pathways under well-watered and drought conditions. (**A**) The heatmap showing the transcript levels of differentially expressed genes (DEGs) in photosynthesis. Data represent the mean value of expression levels after log2 transformation of fragment per kilobase per million reads (FPKM) from triplicate experiments. Each experiment has six plants. Each line in heatmap for one DEG, DEG name and encoded proteins shown at the right side of heatmap, suffix D representing the DEGs from drought conditions, the prefixes 195 and 196 represent the gene belonging to the photosynthesis pathway (map 00195) and antenna protein pathway (map 00196), respectively, in database KEGG (https://www.genome.jp/kegg/); (**B**) DEGs encoding proteins in photosynthesis pathways. Green outbox showing the downregulation of DEGs in sample DP14 relative to D14. The abbreviations in boxes are for DEG encoding proteins, whose information is available at KEGG via https://www.genome.jp/dbget-bin/www_bget?map00195 and https://www.genome.jp/dbget-bin/www_bget?map00196.

To further investigate the roles of DEGs responsible for physiological changes in photosynthesis, we compared the expression levels of induced DEGs across all conditions and annotated DEGs’ functions in photosynthesis. In total, 27 DEGs in photosynthesis antenna protein pathway (KEGG pathway map 00196) and 37 DEGs in photosynthesis pathway (KEGG pathway map 00195) were regulated by BR priming under drought conditions while only one BR-induced DEG with gene id PVE8MA was regulated by BR priming under well-watered conditions (Figure 7), suggesting that expressional regulation in both photo-perception and photosynthesis was from the combined effect of BR-priming and drought. The reduced expression of DEGs, encoding antenna proteins and photosynthesis proteins (Figure 7B), positively correlated with the observed lower photosynthetic rate under drought (Figure 2). The transcript levels of BR-priming induced DEGs in photosynthesis were down-regulated under drought (Figure 7A), suggesting an expressional inhibition. Most DEGs were found between treatments D14 and PD14, which suggested the longer drought induced more DEGs in photosynthesis even with BR priming. Interestingly, two induced DEGs with gene ids T46WBJ and I21UC1 were common in both 7d- and 14 d- drought treatment, and further annotation showed that they are homologous to *Arabidopsis AT1G14150* encoding photosystem II oxygen-evolving enhancer protein 3 (PNSL2) in the electron transport system of photosynthesis II. Expression of both DEGs were downregulated under drought plus BR priming, suggesting consecutively regulation of BR priming on *PNSL2* expression in the drought response. Besides, we observed altered expression levels of DEGs between stages 7d and 14 d even under well-watered conditions, which may indicate an involvement of plant developmental effect, so we used the real-time control for comparison in this study. 

### 3.9. BR Priming Induced Regulation on DEGs in Hormone Signaling Pathways 

**Figure 8:**
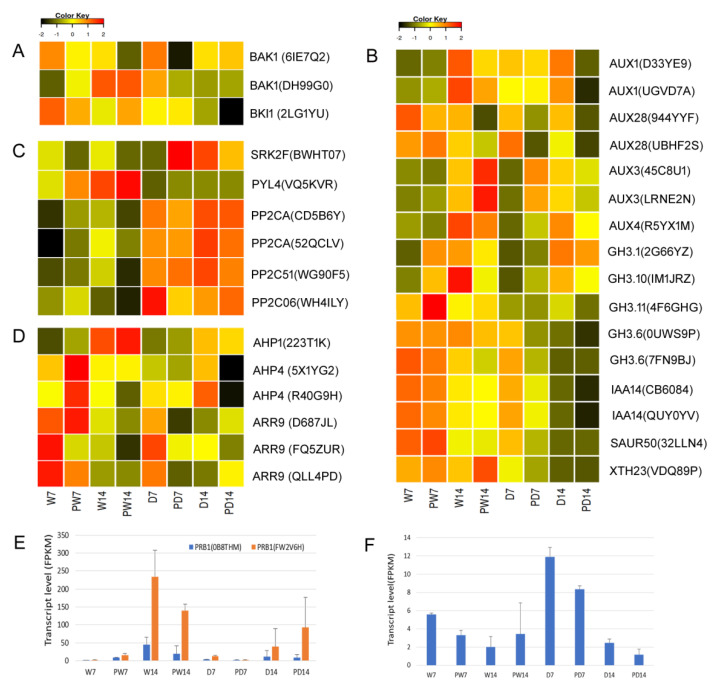
Expression patterns of DEGs in hormone signaling pathways under well-watered and drought conditions. Expression change of differentially expressed genes with at least two-fold changes and *p* < 0.05 in hormone signaling pathways of (**A**) Brassinosteroid (BR), (**B**) auxin, (**C**) abscisic acid (ABA), (**D**) cytokinin, (**E**) salicylic acid (SA), and (**F**) jasmonate (JA). Expression levels were calculated in fragment per kilobase per million reads (FPKM) from triplicate experiments. Heatmap shows the normalized value Z-score after log_2_(FPKM) transformation. W7, PW7, W14, PW14, D7, PD7, D14, and PD14 represents the samples, where 7, 14, W, D, and P represent 7 days of treatment, 14 days of treatment, well-watered condition, drought condition, and seed priming with BR, respectively. The DEG’s id is shown inside a parenthesis. Abbreviations for DEG encoding proteins of AUX/IAA: auxin-responsive protein IAA; GH3: auxin responsive GH3 family; XTH: xyloglucan endotransglucosylase/hydrolases; SAUR: small auxin-up RNA gene family; BAK: BRI1-associated receptor kinase 1; BKI: BRASSINOSTEROID INSENSITIVE 1; SRK2: serine/threonine-protein kinase domain SRK2 subgroup 3; PYL: abscisic acid receptor 1 of PYR/PYL family; PP2C: clade A protein phosphatase 2C; AHP: histidine-containing phosphor-transfer protein; ARR: type-A Arabidopsis response regulator; PRB1: pathogenesis-related protein 1 like; JAZ: JASMONATE ZIM-DOMAIN protein.

To gain more understanding of the regulation resulting from hormone changes, we examined the roles of DEGs and their regulatory patterns in hormone signaling pathways. In total, 38 DEGs were involved in known hormone signaling pathways of ABA, auxin, BR, cytokinin, GA, JA, and SA except the signal pathway of ethylene (Supplementary Table S6). The auxin signaling pathway has the greatest number of DEGs (16 DEGs), followed by six DEGs in ABA signaling pathway. First, we examined the BR-priming effect on BR signal transduction. BRI1-associated receptor kinase 1 (BAK1) and Brassinosteroid receptor BRASSINOSTEROID INSENSITIVE 1 (BRI1) are known to be involved in BR signal which promotes or interferes growth while interacting with environmental conditions [49,50,51]. Here, BR priming induced differentially expressional change of gene *BRI1* and two copies of *BAK1*s. Under well-watered conditions, BR priming induced down regulation of *BAK1* (gene id 6IE7Q2) at stage 14 d and up regulation of another *BAK1* (gene id DH99G0) at stage 7 d, indicating diverse effects on expression of different gene members. Under drought conditions, BR priming induced down regulation of *BAK1* (gene id 6IE7Q2) at stage 7 d, *BAK1* (DH99G0) at stage 14 d, and BKI1 - BRI1 kinase inhibitor 1 (*BKI1*, gene id 2LG1YU) at stage 14 d (Figure 8A), meaning that the BR priming may downregulate the BR perceiving under drought. In the auxin signal pathway, BR priming induced differential expression of multiple copies of gene *AUX/IAA* encoding auxin-responsive protein IAA and gene *GH3* encoding auxin responsive GH3 family. We observed that drought induced down regulation of these DEGs without BR priming. Under water conditions, BR priming induced up regulation of *GH3.1* and *GH3.11* at stage 7 d, down regulation of *AUX1s* and *AUX28s* at stage 14 d, up regulation of *AUX3s* at stage 14 d. Under drought conditions, BR priming induced down regulation of most of DEGs except gene *AUX3s* which were up regulated at stage 7 d (Figure 8B). It is well-known that the ABA signaling pathway is regulated under drought stress [2,3,6]. In the ABA signaling pathway, down regulation, as an expected response, of multiple genes *PP2Cs* encoding clade A protein phosphatase 2C, *SRK2F* or *SnRK2F* encoding serine/threonine-protein kinase domain SRK2 subgroup 3, *PYL4* encoding abscisic acid receptor 1 of PYR/PYL family was identified under drought, but no regulation on *PP2Cs* was found by BR priming compared with those without BR priming. Interestingly, BR priming significantly up regulated expression of *SRK2F* at stage 7 d under drought while the expression was not up regulated under drought compared with under well-watered condition, suggesting BR priming increased the sensitivity of regulation on *SRK2F* under drought (Figure 8C). In cytokinin signal pathway, BR priming induced an up-regulation of two *AHPs* (gene id 5X1YG2 and R40G9H), encoding histidine-containing phosphor-transfer proteins, and *ARR9* (gene id D687JL) encoding type-A *Arabidopsis* response regulators at stage 7 d under well-watered condition, but not at stage 14 d; however, under drought BR priming induced down-regulated expression of *AHPs* and two up-regulated *ARR9s* (gene id D687JL and QLL4PD) out of three ARR9s at stage 7 d (Figure 8D). In the salicylic acid signal pathway, pathogenesis-related protein 1 like (PRB1) is known to be induced in response to pathogen stress [52]. Here, with BR priming, two DEGs encoding PRB1s were up regulated under both well-watered conditions and drought conditions at stage 14 d, suggesting the BR priming induced SA associated immune defense (Figure 8E). In the Jasmonate signal pathway, JASMONATE ZIM-DOMAIN protein (JAZ) regulates the balance between growth and defense. Here, BR priming induced a low *JAZ* expression under drought conditions of both durations 7 d and 14 d (Figure 8F), which may promote growth [53]. 

## 4. Discussion

Drought stress negatively affects terrestrial crop growth and yield worldwide. To date, many efforts have been done to increase drought tolerance or avoid drought [3]. Peanut plants are subject to drought stress which decreased the seed yield. Here we reported the physiological and gene expressional changes resulting from seed priming with brassinolide on peanut plants under drought and well-watered conditions. To our best knowledge, it is the first report with genome-scale transcriptomic analysis of BR seed priming effects on peanut plants. Our results demonstrated that optimal BR priming could reduce partial physiological inhibitions from drought e.g., plant height, but other inhibitions from drought are not rescued, e.g., biomass of peanut plants. With BR priming, the peanut yield components are increased under drought, which may be resulted from BR-priming triggering of increased adaptation to stress and improved late growth after water recovery. This scenario is reported in oak tree with increased growth from re-watering after drought [54]. Brassinosteroids increase grain filling in rice [55]. Therefore, it is possible that BR priming may improve seed filling after water recovery in peanut plants, which will be of high priority in near future investigation. At the gene level, the BR priming induced expressional regulation of many DEGs in well-watered conditions, and fewer DEGs under drought with priming than those under drought without priming. Interestingly, few DEGs induced by drought suggested improved drought tolerance in peanut plants. The scenario is also found in tobacco that a variety with higher drought tolerance has fewer induced DEGs under drought [2]. Besides, DEGs were involved in many enriched GOs and pathways, which indicates an expressional complexity of seed priming of BR. Therefore, our BR-priming DEGs provide clues for further functional investigation of selected genes with BR priming by using overexpression [56] or knock-out expression via CRISPR/Cas9 [56], as our previous report [57]. 

Drought stress inhibits photosynthesis in many plants [3]. Hundreds of genes could be the downstream targets of BR signal after perceived by BRI1 [21]. BR deficiency mutant *pag1* of cotton has drought phenotype and low photosynthetic efficiency [25]. Applied exogenous BR enhanced photosynthesis in cucumber via regulating the photosynthetic enzymes including Rubisco [58]. Transduction of BR signaling could pass from BRI to transcription factors BRASSINAZOLE RESISTANT1 (BZR) and PHYTOCHROME INTERACTING FACTORS (PIFs) to regulate photosynthesis [17]. Thus, the BR priming could transcriptionally regulate photosynthesis in drought stress. Here, our results revealed that BR priming resulted in lower expression of DEGs involved in both light perceiving pathway and photosynthesis pathway under drought than that under drought without priming, which explained the observed lower photosynthesis parameters. Besides, we found that seed priming with BR in peanut is different from directly exogenous BR application on other plants, which increases the photosynthesis e.g., in cucumber [58]. Here BR priming induced increase of chlorophyll, a significant at 0.10 and 0.15 ppm but not statistically significant at 0.20 ppm, which suggested appropriate BR priming concentrations could increase chlorophyll levels but a high concentration may also have adverse role. Seed priming and drought response could be memorized in wheat which enhances tolerance to later occurred salt stress [59]. The drought response could be memorized in some plants [60]. We propose that seed priming with BR will induce a memorized early defense response, which could be recalled and induce a fast response in later occurred stress. This could be via methylation mechanisms at the epigenetic level [10]. In addition, here the observed lower transpiration rates are also associated with BR priming under drought. Low transpiration rate under drought could increase drought tolerance of peanut [14]. Therefore, BR priming induced low photosynthesis and low transpiration could be an indicator of better drought acclimation under stress, which, in return, could increase the final yield in peanut. 

Drought induces changes of hormones in plants. Hormone signals regulate many genes’ expression under drought. Here, drought decreased, as expected, the levels of growth-promoting hormones IAA and GA, as reported previously [2,3,21]. However, BR priming rescued or brought up the hormones, e.g., IAA, to the levels under well-watered conditions. This may explain increased plant height under drought with BR priming. Exogenous BR signal increases auxin biosynthesis and increases expression of the transcription factor genes, e.g., SMALL AUXIN UPREGULATED15 (SAUR15) [17]. Here, we also identified increased *SAUR* expression with BR priming (Figure 8). Therefore, the increased growth could be resulted from the crosstalk between BR signal and auxin signal in BR priming. SA, JA, and ABA regulate plant defense to stress and diseases. Under drought, the significantly increased defense-associated hormones, including SA, JA, and ABA with BR priming compared with those without priming, could trigger the defense to stress. This was demonstrated in the expression patterns of key DEGs in hormone signal pathways. Among the associated DEGs, upregulated *SRK2F* could be the key induced genes in ABA signaling by BR priming. SRK2F could regulate other genes’ expression via kinase cascade [61]. PP2C in the upstream of SRK2F is the down-regulated signal hub under drought [2], however, no effect of BR priming was found on *PP2Cs*, meaning BR priming induced a different regulation on ABA signal at SRK2F instead of PP2C under drought. Interestingly, many copies of DEGs encoding the same protein were identified to be associated with BR priming, e.g., *AUXs* in auxin signal pathway, and *ARRs* in cytokinin signal pathway. Multiple copies of DEGs were found to be regulated at different drought durations, and even different regulatory directions, which could be an adaptation to complicated regulatory networks for responding to varied stresses at different plant developmental stages. Most of DEGs in hormone signal pathways encode kinase or transcription factors [62], which, in return, regulates drought-responsive genes in network manners for better tolerance and adaptation. 

Many known additional regulations in other plants could be also involved in the BR priming, including small RNA regulation [43,63,64,65,66], lncRNA regulation [67], alternative splicing in transcripts [65,68], and methylation regulation [69]. These additional regulations may work in a network manner under drought stress after BR-priming and are worth of dissecting in future using omics methods [70,71]. Investigations of these aspects could further improve understanding of BR-priming mechanism at molecular level. Current finding is focused on the BR priming effects and expressional regulation under drought on one of the most cultivated cultivars. For future investigation, it is valuable to compare the effects of BR priming on more cultivars with different drought responses. For this, a screening of drought responses among germplasm resources will help to identify very high and very low drought responsive variety. Then, it is will be interesting to use the DEGs identified as reference to compare the expressional regulation between selected varieties. A field observation on BR priming effects at a large scale will also provide more clues to narrow down the key regulated genes for improving drought tolerance.

**Figure 9:**
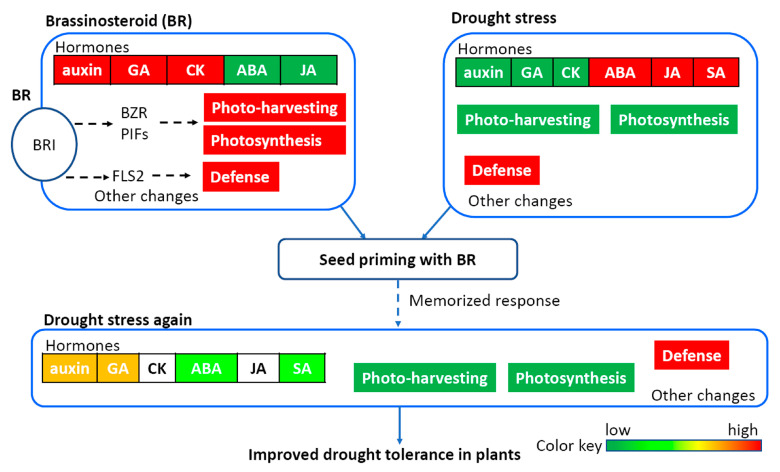
The proposed mechanism of BR priming inducing drought tolerance. The diagram shows the BR priming increases plant growth, photosynthesis, and defense via gene regulation, and the response could be memorized. Once the drought stress occurs, the memorized regulation could be recalled in a fast-adaptive way, which rescues the adverse effects of drought stress. Finally, the seed priming with BR results in an improved drought tolerance in peanut plants. BRI: BRASSINOSTEROID INSENSITIVE1; BZR, BRASSINAZOLE RESISTANT1; PIFs: PHYTOCHROME INTERACTING FACTORS; FLS2: Flagellin sentitive2; ABA: abscisic acid; SA: salicylic acid; GA: gibberellin.

Together, we depict a proposed mechanism of molecular regulation with BR priming after combining the existing knowledge of known BR signal transduction, drought responses, and the finding in this study (Figure 9). After perceiving BR by BRI, the signal is passed into transcription factors including BRASSINAZOLE RESISTANT1 to increase the photosynthesis, growth-promoting hormones, and defense responses. The photosynthesis, growth-promoting hormones, and defense responses are regulated in opposite directions under drought. Once the BR priming responses are induced at the seed stage, those responses may be memorized. The memorized responses may be recalled and induced fast adaptive responses once drought stress occurred. Therefore, the BR priming rescues drought inhibition to bring an improved drought tolerance in peanut plants.

## 5. Conclusions

We investigated the changes of phenotype, physiology, hormones and compared expressional regulation via transcriptome profiling in functional leaves of peanut with/without BR seed priming under drought treatments or well-watered condition. Our study demonstrates that BR seed priming could induce improved drought tolerance in peanut plants and increase yield components via gene expressional regulations in hormones signaling transduction and photosynthesis. We proposed regulatory mechanisms of a fast-adaptive memory recalling from previous BR-priming induced responses in peanut plant when drought stress occurs. Thus, this study improves our understanding of regulatory mechanism of drought response with BR seed priming in peanut.

## Figures and Tables

**Table 1:** The enriched pathways induced by brassinolide priming under drought conditions.

No.	Pathways
1	mRNA surveillance pathway
2	Porphyrin and chlorophyll metabolism
3	Non-homologous end-joining
4	Nitrogen metabolism
5	Proteasome
6	Zeatin biosynthesis
7	RNA polymerase
8	RNA degradation
9	Basal transcription factors
10	Glycosylphosphatidylinositol-anchor biosynthesis

## Data Availability

The RNA-Seq data used in this study are freely available at NCBI short sequence archive under project accession PRJNA629665. All data generated or analyzed during this study are included in the article and its supplementary information files (Supplementary Table S6).

## Supplementary Materials

The following are available online at https://www.mdpi.com/2073-4425/11/8/919/s1, Table S1: The relationship between the SRA accession number, treatments, and data in NCBI. Table S2: Effects of seed priming with brassinolide on peanut plant height and ratio under well-watered and drought conditions. Table S3: Effects of BR priming on biomass of peanut plants under well-watered and drought conditions. Table S4: Effects of BR priming on peanut yield components under well-watered and drought conditions. Table S5: Effects of BR priming on kernel quality of peanut under well-watered and drought conditions. Table S6: Differentially expressed genes in plant hormone signaling pathways.

## References

[1] Burgess P., Huang B., Hossain M.A., Wani S.H., Bhattacharjee S., Burritt D.J., Tran L.-S.P. (2016). Mechanisms of hormone regulation for drought tolerance in plants. Drought Stress Tolerance in Plants, Vol 1: Physiology and Biochemistry.

[2] Wang J., Zhang S., Fu Y., He T., Wang X. (2020). Analysis of dynamic global transcriptional atlas reveals common regulatory networks of hormones and photosynthesis across nicotiana varieties in response to long-term drought. Front. Plant Sci..

[3] Shanker A.K., Maheswari M., Yadav S.K., Desai S., Bhanu D., Attal N.B., Venkateswarlu B. (2014). Drought stress responses in crops. Funct. Integr. Genom..

[4] Basu S., Ramegowda V., Kumar A., Pereira A. (2016). Plant adaptation to drought stress. F1000Research.

[5] Zargar S.M., Gupta N., Nazir M., Mahajan R., Malik F.A., Sofi N.R., Shikari A.B., Salgotra R.K. (2017). Impact of drought on photosynthesis: Molecular perspective. Plant Gene.

[6] Mega R., Abe F., Kim J.-S., Tsuboi Y., Tanaka K., Kobayashi H., Sakata Y., Hanada K., Tsujimoto H., Kikuchi J. (2019). Tuning water-use efficiency and drought tolerance in wheat using abscisic acid receptors. Nat. Plants.

[7] Chai C., Subudhi P.K. (2016). Comprehensive analysis and expression profiling of the OsLAX and OsABCB auxin transporter gene families in rice (Oryza sativa) under phytohormone stimuli and abiotic stresses. Front. Plant Sci..

[8] Korver R.A., Koevoets I.T., Testerink C. (2018). Out of shape during stress: A key role for auxin. Trends Plant Sci..

[9] Gupta A., Rico-Medina A., Caño-Delgado A.I. (2020). The physiology of plant responses to drought. Science.

[10] Mateo-Bonmatí E., Casanova-Sáez R., Ljung K. (2019). Epigenetic regulation of auxin homeostasis. Biomolecules.

[11] Wang X., Liu F.-l., Jiang D. (2017). Priming: A promising strategy for crop production in response to future climate. J. Integr. Agric..

[12] Hussain S., Zheng M., Khan F., Khaliq A., Fahad S., Peng S., Huang J., Cui K., Nie L. (2015). Benefits of rice seed priming are offset permanently by prolonged storage and the storage conditions. Sci. Rep..

[13] Farooq M., Basra S., Hafeez K. (2006). Seed invigoration by osmohardening in coarse and fine rice. Seed Sci. Technol..

[14] Paparella S., Araújo S., Rossi G., Wijayasinghe M., Carbonera D., Balestrazzi A. (2015). Seed priming: State of the art and new perspectives. Plant Cell Rep..

[15] Jisha K., Vijayakumari K., Puthur J.T. (2013). Seed priming for abiotic stress tolerance: An overview. Acta Physiol. Plant..

[16] Brocklehurst P., Dearman J. (1983). Interactions between seed priming treatments and nine seed lots of carrot, celery and onion. II. Seedling emergence and plant growth. Ann. Appl. Biol..

[17] Sneideris L.C., Gavassi M.A., Campos M.L., D’Amico-Damiao V., Carvalho R.F. (2015). Effects of hormonal priming on seed germination of pigeon pea under cadmium stress. An. Acad. Bras. Ciências.

[18] Gao Y.-P., Bonham-Smith P.C., Gusta L.V. (2002). The role of peroxiredoxin antioxidant and calmodulin in ABA-primed seeds of *Brassica napus* exposed to abiotic stresses during germination. J. Plant Physiol..

[19] Chung Y., Choe S. (2013). The regulation of brassinosteroid biosynthesis in Arabidopsis. Crit. Rev. Plant Sci..

[20] Ahammed G.J., Li X., Liu A., Chen S. (2020). Brassinosteroids in plant tolerance to abiotic stress. J. Plant Growth Regul..

[21] Zhu J., Sae-Seaw J., Wang Z. (2013). Brassinosteroid signalling. Development.

[22] Yang P., Wang Y., Li J., Bian Z. (2019). Effects of brassinosteroids on photosynthetic performance and nitrogen metabolism in pepper seedlings under chilling stress. Agronomy.

[23] Soares T.F.S.N., Dias D.C.F.D.S., Oliveira A.M.S., Ribeiro D.M., Dias L.A.D.S. (2020). Exogenous brassinosteroids increase lead stress tolerance in seed germination and seedling growth of *Brassica juncea* L.. Ecotoxicol. Environ. Saf..

[24] Zhao M., Yuan L., Wang J., Xie S., Zheng Y., Nie L., Zhu S., Hou J., Chen G., Wang C. (2019). Transcriptome analysis reveals a positive effect of brassinosteroids on the photosynthetic capacity of wucai under low temperature. BMC Genom..

[25] Chen E., Zhang X., Yang Z., Zhang C., Wang X., Ge X., Li F. (2019). BR deficiency causes increased sensitivity to drought and yield penalty in cotton. BMC Plant Biol..

[26] Lee J., Shim D., Moon S., Kim H., Bae W., Kim K., Kim Y.H., Rhee S.K., Hong C.P., Hong S.Y. (2018). Genome-wide transcriptomic analysis of BR-deficient Micro-Tom reveals correlations between drought stress tolerance and brassinosteroid signaling in tomato. Plant Physiol. Biochem..

[27] Anuradha S., Ram Rao S.S. (2003). Application of brassinosteroids to rice seeds (*Oryza sativa* L.) reduced the impact of salt stress on growth, prevented photosynthetic pigment loss and increased nitrate reductase activity. Plant Growth Regul..

[28] Zhang S., Hu J., Zhang Y., Xie X., Knapp A. (2007). Seed priming with brassinolide improves lucerne (*Medicago sativa* L.) seed germination and seedling growth in relation to physiological changes under salinity stress. Aust. J. Agric. Res..

[29] Reddy T., Reddy V., Anbumozhi V. (2003). Physiological responses of groundnut (*Arachis hypogea* L.) to drought stress and its amelioration: A critical review. Plant Growth Regul..

[30] Sinclair T.R., Shekoofa A., Isleib T.G., Balota M., Zhang H. (2018). Identification of Virginia-Type Peanut Genotypes for Water-Deficit Conditions Based on Early Decrease in Transpiration Rate with Soil Drying. Crop Sci..

[31] Bertioli D.J., Cannon S.B., Froenicke L., Huang G., Farmer A.D., Cannon E.K.S., Liu X., Gao D., Clevenger J., Dash S. (2016). The genome sequences of Arachis duranensis and Arachis ipaensis, the diploid ancestors of cultivated peanut. Nat. Genet..

[32] Chen X., Li H., Pandey M.K., Yang Q., Wang X., Garg V., Li H., Chi X., Doddamani D., Hong Y. (2016). Draft genome of the peanut A-genome progenitor (Arachis duranensis) provides insights into geocarpy, oil biosynthesis, and allergens. Proc. Natl. Acad. Sci. USA.

[33] Zhuang W., Chen H., Yang M., Wang J., Pandey M.K., Zhang C., Chang W., Zhang L., Zhang X., Tang R. (2019). The genome of cultivated peanut provides insight into legume karyotypes, polyploid evolution and crop domestication. Nat. Genet..

[34] Bertioli D.J., Jenkins J., Clevenger J., Dudchenko O., Gao D., Seijo G., Leal-Bertioli S.C.M., Ren L., Farmer A.D., Pandey M.K. (2019). The genome sequence of segmental allotetraploid peanut Arachis hypogaea. Nat. Genet..

[35] Chen X., Lu Q., Liu H., Zhang J., Hong Y., Lan H., Li H., Wang J., Liu H., Li S. (2019). Sequencing of cultivated peanut, Arachis hypogaea, yields insights into genome evolution and oil improvement. Mol. Plant.

[36] Arunyanark A., Pimratch S., Jogloy S., Wongkaew S., Vorasoot N., Akkasaeng C., Kesmala T., Patanothai A., Holbrook C. (2012). Association between aflatoxin contamination and N2 fixationin peanut under drought conditions. Int. J. Plant Prod..

[37] Songsri P., Jogloy S., Vorasoot N., Akkasaeng C., Patanothai A., Holbrook C. (2008). Root distribution of drought-resistant peanut genotypes in response to drought. J. Agron. Crop Sci..

[38] Liang J., Zhang J., Wong M.H. (1997). Can stomatal closure caused by xylem ABA explain the inhibition of leaf photosynthesis under soil drying?. Photosynth. Res..

[39] Liu R.-X., Zhou Z.-G., Guo W.-Q., Chen B.-L., Oosterhuis D.M. (2008). Effects of N fertilization on root development and activity of water-stressed cotton (Gossypium hirsutum L.) plants. Agric. Water Manag..

[40] Weng Y., Shi A., Ravelombola W.S., Yang W., Qin J., Motes D., Moseley D.O., Chen P. (2017). A rapid method for measuring seed protein content in cowpea (*Vigna unguiculata* (L.) Walp). Am. J. Plant Sci..

[41] Zhang G.-C., Dai L.-X., Ding H., Ci D.-W., Ning T.-Y., Yang J.-S., Zhao X.-H., Yu H.-Q., Zhang Z.-M. (2020). Response and adaptation to the accumulation and distribution of photosynthetic product in peanut under salt stress. J. Integr. Agric..

[42] Pan X., Welti R., Wang X. (2010). Quantitative analysis of major plant hormones in crude plant extracts by high-performance liquid chromatography-mass spectrometry. Nat. Protoc..

[43] Chen Y., Dong J., Bennetzen J.L., Zhong M., Yang J., Zhang J., Li S., Hao X., Zhang Z., Wang X. (2017). Integrating transcriptome and microRNA analysis identifies genes and microRNAs for AHO-induced systemic acquired resistance in N. tabacum. Sci. Rep..

[44] Wang X., Liu B.-Y., Zhao Q., Sun X., Li Y., Duan Z., Miao X., Luo S., Li J. (2019). Genomic Variance and Transcriptional Comparisons Reveal the Mechanisms of Leaf Color Affecting Palatability and Stressed Defense in Tea Plant. Genes.

[45] Moriya Y., Itoh M., Okuda S., Yoshizawa A.C., Kanehisa M. (2007). KAAS: An automatic genome annotation and pathway reconstruction server. Nucleic Acids Res..

[46] Pertea M., Kim D., Pertea G.M., Leek J.T., Salzberg S.L. (2016). Transcript-level expression analysis of RNA-seq experiments with HISAT, StringTie and Ballgown. Nat. Protoc..

[47] Love M.I., Huber W., Anders S. (2014). Moderated estimation of fold change and dispersion for RNA-seq data with DESeq2. Genome Biol..

[48] Clevenger J., Chu Y., Scheffler B., Ozias-Akins P. (2016). A developmental transcriptome map for allotetraploid Arachis hypogaea. Front. Plant Sci..

[49] Sun Y., Han Z., Tang J., Hu Z., Chai C., Zhou B., Chai J. (2013). Structure reveals that BAK1 as a co-receptor recognizes the BRI1-bound brassinolide. Cell Res..

[50] Peng Y., Chen L., Li S., Zhang Y., Xu R., Liu Z., Liu W., Kong J., Huang X., Wang Y. (2018). BRI1 and BAK1 interact with G proteins and regulate sugar-responsive growth and development in Arabidopsis. Nat. Commun..

[51] Belkhadir Y., Jaillais Y. (2015). The molecular circuitry of brassinosteroid signaling. New Phytol..

[52] Ali S., Ganai B.A., Kamili A.N., Bhat A.A., Mir Z.A., Bhat J.A., Tyagi A., Islam S.T., Mushtaq M., Yadav P. (2018). Pathogenesis-related proteins and peptides as promising tools for engineering plants with multiple stress tolerance. Microbiol. Res..

[53] Guo Q., Yoshida Y., Major I.T., Wang K., Sugimoto K., Kapali G., Havko N.E., Benning C., Howe G.A. (2018). JAZ repressors of metabolic defense promote growth and reproductive fitness in *Arabidopsis*. Proc. Natl. Acad. Sci. USA.

[54] Spieß N., Oufir M., Matušíková I., Stierschneider M., Kopecky D., Homolka A., Burg K., Fluch S., Hausman J.-F., Wilhelm E. (2012). Ecophysiological and transcriptomic responses of oak (*Quercus robur*) to long-term drought exposure and rewatering. Environ. Exp. Bot..

[55] Wu C.-Y., Trieu A., Radhakrishnan P., Kwok S.F., Harris S., Zhang K., Wang J., Wan J., Zhai H., Takatsuto S. (2008). Brassinosteroids regulate grain filling in rice. Plant Cell.

[56] Prelich G. (2012). Gene Overexpression: Uses, Mechanisms, and Interpretation. Genetics.

[57] Zhou Y., Zhang Y., Wang X., Han X., An Y., Lin S., Shen C., Wen J., Liu C., Yin W. (2020). Root-specific NF-Y family transcription factor, PdNF-YB21, positively regulates root growth and drought resistance by abscisic acid-mediated indoylacetic acid transport in Populus. New Phytol..

[58] Xia X.-J., Huang L.-F., Zhou Y.-H., Mao W.-H., Shi K., Wu J.-X., Asami T., Chen Z., Yu J.-Q. (2009). Brassinosteroids promote photosynthesis and growth by enhancing activation of Rubisco and expression of photosynthetic genes in Cucumis sativus. Planta.

[59] Tabassum T., Farooq M., Ahmad R., Zohaib A., Wahid A. (2017). Seed priming and transgenerational drought memory improves tolerance against salt stress in bread wheat. Plant Physiol. Biochem..

[60] Walter J., Nagy L., Hein R., Rascher U., Beierkuhnlein C., Willner E., Jentsch A. (2011). Do plants remember drought? Hints towards a drought-memory in grasses. Environ. Exp. Bot..

[61] Joshi-Saha A., Valon C., Leung J. (2011). Abscisic Acid Signal off the STARTing Block. Mol. Plant.

[62] McSteen P., Zhao Y. (2008). Plant Hormones and Signaling: Common Themes and New Developments. Dev. Cell.

[63] Liu S., Mi X., Zhang R., An Y., Zhou Q., Yang T., Xia X., Guo R., Wang X., Wei C. (2019). Integrated analysis of miRNAs and their targets reveals that miR319c/TCP2 regulates apical bud burst in tea plant (*Camellia sinensis*). Planta.

[64] Jeyaraj A., Wang X., Wang S., Liu S., Zhang R., Wu A., Wei C. (2019). Identification of Regulatory Networks of MicroRNAs and Their Targets in Response to Colletotrichum gloeosporioides in Tea Plant (*Camellia sinensis* L.). Front. Plant Sci..

[65] Zhu J., Wang X., Xu Q., Zhao S., Tai Y., Wei C. (2018). Global dissection of alternative splicing uncovers transcriptional diversity in tissues and associates with the flavonoid pathway in tea plant (*Camellia sinensis*). BMC Plant Biol..

[66] Zhao S., Wang X., Yan X., Guo L., Mi X., Xu Q., Zhu J., Wu A., Liu L., Wei C. (2018). Revealing of MicroRNA Involved Regulatory Gene Networks on Terpenoid Biosynthesis in *Camellia sinensis* in Different Growing Time Points. J. Agric. Food Chem..

[67] Han G., Cheng C., Zheng Y., Wang X., Xu Y., Wang W., Zhu S., Cheng B. (2019). Identification of Long Non-Coding RNAs and the Regulatory Network Responsive to Arbuscular Mycorrhizal Fungi Colonization in Maize Roots. Int. J. Mol. Sci..

[68] Thatcher S.R., Danilevskaya O.N., Meng X., Beatty M., Zastrow-Hayes G., Harris C., Van Allen B., Habben J., Li B. (2016). Genome-Wide Analysis of Alternative Splicing during Development and Drought Stress in Maize. Plant Physiol..

[69] Thiebaut F., Hemerly A.S., Ferreira P.C.G. (2019). A Role for Epigenetic Regulation in the Adaptation and Stress Responses of Non-model Plants. Front. Plant Sci..

[70] Zhang S., Zhang L., Tai Y., Wang X., Ho C.-T., Wan X. (2018). Gene Discovery of Characteristic Metabolic Pathways in the Tea Plant (*Camellia sinensis*) Using ‘Omics’-Based Network Approaches: A Future Perspective. Front. Plant Sci..

[71] Gupta B., Sengupta A., Saha J., Gupta K. (2013). Plant Abiotic Stress: OMICS Approach. J. Plant Biochem. Physiol..

