# Supplementary material for: Brassinosteroid Priming Improves Peanut Drought Tolerance via Eliminating Inhibition on Genes in Photosynthesis and Hormone Signaling
Source: Genes (Basel). 2020 Aug 11;11(8):919. doi: 10.3390/genes11080919 (PMC7465412; doi:10.3390/genes11080919)
Supplement: Supplementary file 1 [file genes-11-00919-s001.pdf]

Table 1. The relationship between the SRA accession number, treatments and data in NCBI.

SRA number	Treatments#	Data name
**SRR11659571**	W7-1	R1908096_BKDL190834194-1a_1.fq.gz, R1908096_BKDL190834194-1a_2.fq.gz
SRR11659570	W7-2	R1908097_BKDL190834195-1a_1.fq.gz, R1908097_BKDL190834195-1a_2.fq.gz
SRR11659569	W7-3	R1908098_BKDL190834196-1a_1.fq.gz, R1908098_BKDL190834196-1a_2.fq.gz
SRR11659564	PW7-1	R1908102_BKDL190834200-1a_1.fq.gz, R1908102_BKDL190834200-1a_2.fq.gz
SRR11659563	PW7-2	R1908103_BKDL190834201-1a_1.fq.gz, R1908103_BKDL190834201-1a_2.fq.gz
SRR11659562	PW7-3	R1908104_BKDL190834202-1a_1.fq.gz, R1908104_BKDL190834202-1a_2.fq.gz
SRR11659568	D7-1	R1908099_BKDL190834197-1a_1.fq.gz, R1908099_BKDL190834197-1a_2.fq.gz
SRR11659567	D7-2	R1908100_BKDL190834198-1a_1.fq.gz, R1908100_BKDL190834198-1a_2.fq.gz
SRR11659565	D7-3	R1908101_BKDL190834199-1a_1.fq.gz, R1908101_BKDL190834199-1a_2.fq.gz
SRR11659561	PD7-1	R1908105_BKDL190834203-1a_1.fq.gz, R1908105_BKDL190834203-1a_2.fq.gz
SRR11659560	PD7-2	R1908106_BKDL190834204-1a_1.fq.gz, R1908106_BKDL190834204-1a_2.fq.gz
SRR11659559	PD7-3	R1908107_BKDL190834205-1a_1.fq.gz, R1908107_BKDL190834205-1a_2.fq.gz
SRR11659558	W14-1	R1908108_BKDL190834206-1a_1.fq.gz, R1908108_BKDL190834206-1a_2.fq.gz
SRR11659557	W14-2	R1908109_BKDL190834207-1a_1.fq.gz, R1908109_BKDL190834207-1a_2.fq.gz
SRR11659556	W14-3	R1908110_BKDL190834208-1a_1.fq.gz, R1908110_BKDL190834208-1a_2.fq.gz
SRR11659551	PW14-1	R1908114_BKDL190834212-1a_1.fq.gz, R1908114_BKDL190834212-1a_2.fq.gz
SRR11659550	PW14-2	R1908115_BKDL190834213-1a_1.fq.gz, R1908115_BKDL190834213-1a_2.fq.gz
SRR11659549	PW14-3	R1908116_BKDL190834214-1a_1.fq.gz, R1908116_BKDL190834214-1a_2.fq.gz
SRR11659554	D14-1	R1908111_BKDL190834209-1a_1.fq.gz, R1908111_BKDL190834209-1a_2.fq.gz
SRR11659553	D14-2	R1908112_BKDL190834210-1a_1.fq.gz, R1908112_BKDL190834210-1a_2.fq.gz
SRR11659552	D14-3	R1908113_BKDL190834211-1a_1.fq.gz, R1908113_BKDL190834211-1a_2.fq.gz
SRR11659548	PD14-1	R1908117_BKDL190834215-1a_1.fq.gz, R1908117_BKDL190834215-1a_2.fq.gz
SRR11659547	PD14-2	R1908118_BKDL190834216-1a_1.fq.gz, R1908118_BKDL190834216-1a_2.fq.gz
SRR11659546	PD14-3	R1908119_BKDL190834217-1a_1.fq.gz, R1908119_BKDL190834217-1a_2.fq.gz

Note: -1, -2, -3 for replicates.

Table 2. Effects of seed priming with brassinolide on plant height of peanut under well-watered and drought conditions.

BR concentrations (ppm)		Well-watered	Drought	Ratio of Drought/Well-watered	Ratio of priming/no priming under well-watered	Ratio of priming/no priming under drought
7 days	0.00	18.35±0.14b	12.93±0.96b*	70.46±5.82b	--	--
	0.05	15.81±1.56b	12.72±1.19b	80.42±0.39ab	86.16±7.85b	99.00±16.67a
	0.10	22.08±2.00a	17.64±0.39a	80.30±9.10ab	120.29±10.02a	146.40±6.46a
	0.15	19.13±1.11ab	18.45±1.27a	96.83±12.35a	104.23±5.29ab	125.71±24.99a
	0.20	16.67±0.47b	14.93±0.11b*	89.61±3.19ab	90.84±1.84b	99.43±30.97a
14 days	0.00	19.25±0.12b	16.55±1.47ab	85.92±7.11a	--	--
	0.05	16.92±1.83b	13.58±1.03b	80.42±2.60a	87.90±10.10b	82.69±13.63a
	0.10	25.60±1.00a	14.61±1.40b*	57.24±7.74b	132.97±4.34a	89.07±16.46a
	0.15	25.99±1.78a	19.32±1.26a*	74.32±0.23a	135.05±10.15a	117.55±18.15a
	0.20	20.00±3.76b	15.19±1.15b	76.75±8.66a	103.99±20.27ab	92.49±15.27a

Note: Data represent mean ± standard error; Letters after data represent statistical significance at $p < 0.05$ with Least Significant Difference (LSD) test. * represent statistically significant difference ($p < 0.05$) between well-watered and drought conditions.

Table 3. Effects of BR priming on biomass of peanut plants under well-watered and drought conditions.

BR concentrations(ppm)		DWR (g)		DWS (g)		DWL (g)	
		**Well-watered**	**Drought**	**Well-watered**	**Drought**	**Well-watered**	**Drought**
7 days	0.00	0.25±0.01b	0.22±0.02b	1.20±0.08ab	0.86±0.09b	2.11±0.03ab	1.75±0.07c
	0.05	0.28±0.01b	0.24±0.01ab	1.10±0.14ab	0.89±0.19b	2.23±0.27ab	1.95±0.04b
	0.10	0.34±0.01a	0.26±0.01a	1.23±0.07a	1.10±0.04a	2.55±0.01a	2.30±0.06a
	0.15	0.35±0.01a	0.28±0.01a	1.36±0.10a	1.20±0.07a	2.22±0.25ab	2.25±0.19a
	0.20	0.25±0.02b	0.23±0.02b	1.01±0.05b	0.83±0.19b	1.76±0.10b	1.63±0.18c
14 days	0.00	0.48±0.02a	0.34±0.02b	2.81±0.14bc	1.64±0.07b	4.13±0.37b	2.69±0.10b
	0.05	0.42±0.01ab	0.29±0.01b	2.86±0.26bc	1.76±0.09b	4.47±0.21b	2.53±0.29b
	0.10	0.44±0.05ab	0.38±0.01ab	3.04±0.08a	1.81±0.11b	5.33±0.04a*	3.07±0.18a
	0.15	0.51±0.02a	0.41±0.03a	3.06±0.18a*	2.05±0.11a	5.34±0.06a*	3.14±0.29a
	0.20	0.32±0.01b	0.27±0.01b	2.64±0.21c	1.42±0.04b	3.86±0.36b	2.05±0.09c

Note: Data represent mean ± standard error; Letters after value represent statistical significance at $p < 0.05$ with Least significant Difference (LSD) test. * represent statistically significant difference ($p < 0.05$) between well-watered and drought conditions. DWR, DWS and DWL represent dry weight of roots, stems, and leaves.

Table 4. Effects of BR priming on peanut yield components under well-watered and drought conditions.

BR concentrations (ppm)		Number of pods per plant	Hundred pod weight (g)	Hundred kernel weight (g)	Shelling rate (%)
0.00 ppm	Well-watered	9.6±1.67a	75.8±1.07a	30.8±0.43a	71.6±1.01a
	7d drought	10.0±0.00a	67.5±0.95b	27.9±0.30b	71.9±1.02a
	14d drought	7.6±1.14b	59.2±0.88c	24.6±0.34c	71.6±0.91a
0.15 ppm	Well-watered	14.5±1.64a**	85.8b±1.21b**	39.2b±0.55b**	77.0±1.09a**
	7d drought	15.3±2.08a**	97.4a±1.38a**	43.0a±0.61a**	74.5±0.85b*
	14d drought	9.6±1.52b*	63.25±0.89c*	27.6±0.39c*	74.1±1.05b*

Note: Data are presented as the mean ± standard error (n =3). Letters after the value represent the statistically significant difference between each BR concentration ($p < 0.05$) as determined by the LSD test. * and ** represent the statistically significant difference at $p < 0.05$ and $p < 0.01$, respectively, between 0.15 ppm BR priming and 0 ppm BR priming. 7d, 7 days; 14d, 14 days.

Table 5. Effects of BR priming on kernel quality of peanut under well-watered and drought conditions.

BR treatments		Oil content (%)	Protein content (%)
0.00 ppm	Well-watered	50.79±0.13ab	28.13±0.12b
	7d drought	51.01±0.50a	29.37±0.21a
	14d drought	49.53±0.47b	27.90±0.19b
0.15 ppm	Well-watered	52.96±0.20a	28.95±0.70c
	7d drought	51.60±0.41b	30.95±0.84a
	14d drought	50.30±0.11c	29.97±0.68b

Note: Data are expressed as the mean ± S.E. (n = 3). Different letters within each growth stage are significantly different ($P < 0.05$) as determined by the LSD test. * represents the comparison between 0.15 ppm with 0 ppm.

Table 6. Differentially expressed genes in plant hormone signaling pathways.

DEG id in peanut genome	Name in pathway*	Associated hormone
arahy.Tifrunner.gnm1.ann1.WH4ILY	PP2C06	ABA
arahy.Tifrunner.gnm1.ann1.WG90F5	PP2C51	ABA
arahy.Tifrunner.gnm1.ann1.52QCLV	PP2CA	ABA
arahy.Tifrunner.gnm1.ann1.CD5B6Y	PP2CA	ABA
arahy.Tifrunner.gnm1.ann1.VQ5KVR	PYL4	ABA
arahy.Tifrunner.gnm1.ann1.BWHT07	SRK2F	ABA
arahy.Tifrunner.gnm1.ann1.944YYF	AUX28	auxin
arahy.Tifrunner.gnm1.ann1.UBHF2S	AUX28	auxin
arahy.Tifrunner.gnm1.ann1.2G66YZ	GH3.1	auxin
arahy.Tifrunner.gnm1.ann1.IM1JRZ	GH3.10	auxin
arahy.Tifrunner.gnm1.ann1.4F6GHG	GH3.11	auxin
arahy.Tifrunner.gnm1.ann1.0UWS9P	GH3.6	auxin
arahy.Tifrunner.gnm1.ann1.7FN9BJ	GH3.6	auxin
arahy.Tifrunner.gnm1.ann1.CB6084	IAA14	auxin
arahy.Tifrunner.gnm1.ann1.QUY0YV	IAA14	auxin
arahy.Tifrunner.gnm1.ann1.D33YE9	AUX1	auxin
arahy.Tifrunner.gnm1.ann1.UGVD7A	AUX1	auxin
arahy.Tifrunner.gnm1.ann1.45C8U1	AUX3	auxin
arahy.Tifrunner.gnm1.ann1.LRNE2N	AUX3	auxin
arahy.Tifrunner.gnm1.ann1.R5YX1M	AUX4	auxin
arahy.Tifrunner.gnm1.ann1.32LLN4	SAUR50	auxin
arahy.Tifrunner.gnm1.ann1.VDQ89P	XTH23	auxin
arahy.Tifrunner.gnm1.ann1.6IE7Q2	BAK1	BR
arahy.Tifrunner.gnm1.ann1.DH99G0	BAK1	BR
arahy.Tifrunner.gnm1.ann1.2LG1YU	BKI1	BR
arahy.Tifrunner.gnm1.ann1.R40G9H	AHP4	Cytokinin
arahy.Tifrunner.gnm1.ann1.D687JL	ARR9	Cytokinin
arahy.Tifrunner.gnm1.ann1.223T1K	AHP1	Cytokinin
arahy.Tifrunner.gnm1.ann1.5X1YG2	AHP4	Cytokinin
arahy.Tifrunner.gnm1.ann1.FQ5ZUR	ARR9	Cytokinin
arahy.Tifrunner.gnm1.ann1.QLL4PD	ARR9	Cytokinin
arahy.Tifrunner.gnm1.ann1.99WGGU	GID1B	GA
arahy.Tifrunner.gnm1.ann1.4I27IK	PIF4	GA
arahy.Tifrunner.gnm1.ann1.QKV9FM	PIL15	GA
arahy.Tifrunner.gnm1.ann1.TPQ86D	JAZ	JA
arahy.Tifrunner.gnm1.ann1.0B8THM	PRB1	SA
arahy.Tifrunner.gnm1.ann1.FW2V6H	PRB1	SA
arahy.Tifrunner.gnm1.ann1.W4QI9P	TGA10	SA

*Note: the pathway is referred to as KEGG plant hormone signaling pathway (id ath04075), accessed on April 29th, 2020.
